# Structural modulation of oleogel-based ice cream: Effect of ultrasonically treated whey protein isolate fibrils on fat coalescence and aeration properties

**DOI:** 10.1016/j.ultsonch.2026.108069

**Published:** 2026-09-19

**Authors:** Ning Liu, Meng Yan, Junqi Sha, Kun Zhang, Xiaoting Xue, Wenguang Liu, Xiaomiao Jing, Xiaolin Yao

**Affiliations:** a School of Food Science and Engineering, Shaanxi University of Science & Technology, Xi’an 710021, China; b Shenguan Holdings (Group) Ltd., Wuzhou 543000, China; c Ferguson (Wuhan) Biotechnology Co., Ltd., Wuhan 430056, China

**Keywords:** Whey protein isolate fibril, Ultrasonication, Ice cream, Fat coalescence, Oral tribology

## Abstract

The effect of ultrasonically treated whey protein isolate fibrils (WPIFs) on fat coalescence and aeration properties of oleogel (OG)-based ice cream was investigated. An 8-minute ultrasonication treatment at 500 W and 20 kHz facilitated the fragmentation of WPIF into short fibrils with an average length of 225.88 ± 22.75 nm. This procedure increased the exposure of hydrophobic residues (H_0_ = 262.43 ± 1.14) and improved interfacial adsorption. The incorporation of modified WPIFs into the ice cream emulsion promoted the formation of denser and smaller fat and ice crystals, and enhanced the viscoelastic properties. The WPIF-8-OG ice cream demonstrated a partial coalescence degree of 217.60 ± 7.62%, an overrun of 70.63 ± 0.92%, and a hardness of 1474.10 ± 21.62 g. This sample exhibited favorable lubrication properties in oral tribological analysis, along with improved melting behavior and air bubble distribution. However, ultrasonication treatment for 12 min induced irregular ice crystal formation and compromised the viscoelastic properties of emulsion, thereby increasing the frictional force observed in ice cream. These findings provide valuable insights for the design strategies of OG-based frozen dairy formulations.

## Highlights

• Controlled ultrasonication resulted in uniform whey protein isolate fibrils (WPIFs).
• WPIF promoted even distribution of fat and ice crystals in oleogel-based emulsion.
• WPIF-8 enhanced the texture, overrun, and tribological properties of ice cream.
• Oleogel-based ice creams exhibited improved melting behavior and bubble formation.

## Graphical abstract

**Figure:**
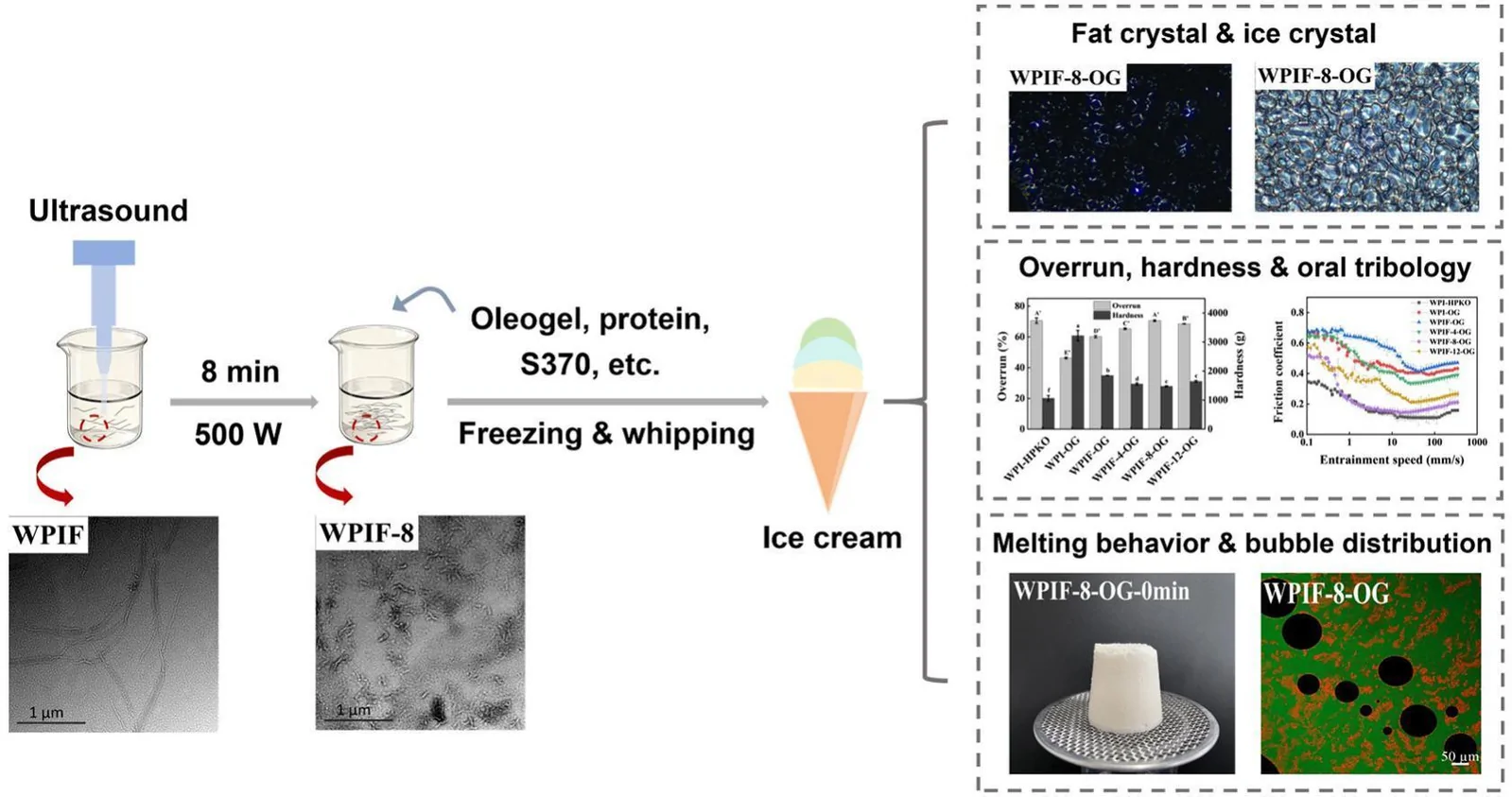

## 1 Introduction

Ice cream is a widely consumed frozen dairy product that constitutes a complex colloidal system with ice crystals, air bubbles, and partially coalesced fat droplets. The structural characteristics of ice cream are significantly influenced by its ingredient composition, with fat playing a critical role in stabilizing ice crystals, enhancing aeration, improving melt resistance, and contributing to both texture and flavor profiles [1]. Typically, the fat content in ice cream ranges between 10% and 18%, predominantly derived from butterfat, cream, and vegetable fats [2]. The direct replacement of saturated fats with unsaturated oils presents challenges, as it often compromises the structural integrity and sensory qualities of ice cream, largely due to the critical role of the fat crystal network.

Oleogels (OGs), formed by incorporating gelators such as beeswax, β-sitosterol + γ-oryzanol, or ethyl cellulose into liquid oils, have emerged as promising alternatives to traditional solid fats. The crystalline or polysaccharide networks inherent to OGs functionally mimic those of solid fats while offering improved nutritional profiles through reduced saturated fatty acid content [3]. A study has demonstrated that the application of beeswax-based oleogels increased the proportion of unsaturated fatty acids, enhanced the stability and texture of ice cream, and improved its shape retention during melting [4].

Proteins play a significant role in the structural integrity of ice cream by facilitating emulsification, aeration, and water retention. Adsorbed proteins contribute to the stabilization of fat globules and air bubbles during homogenization and whipping processes, respectively, whereas non-adsorbed proteins increase viscosity and inhibit the growth of ice crystals [5]. Whey protein isolates (WPI) are extensively employed due to their multifunctional properties, including emulsification and foam stabilization [6]. The conformational flexibility of proteins affects their interfacial behavior: proteins with random-coil structures rapidly adsorb at oil–water interfaces, while globular proteins necessitate structural rearrangements to expose hydrophobic residues [7]. Functional enhancements of proteins can be achieved through modifications such as thermal treatment, enzymatic crosslinking, or fibrillation. Thermally induced aggregation of WPI can emulate the behavior of casein micelles, thereby improving the freeze–thaw stability of emulsions [8].

Protein fibrillation, typically induced by acidic heating, results in the formation of amyloid-like fibrils characterized by β-sheet structures. These fibrils are non-toxic and have been applied in emulsions, foams, and hydrogels due to their capacity to form robust interfacial films [9]. WPI fibrils (WPIFs) are typically prepared by heating WPI above 80 ℃ for several hours (5–24 h) under acidic conditions and low ionic strength. WPIF can generate smaller and more uniform emulsion droplets and reduce lipid oxidation compared to native WPI [10]. The stability of emulsions is significantly influenced by the length and morphology of fibrils, wherein shorter fibrils play a crucial role in reducing bridging flocculation. WPIF has exhibited potential as an inhibitor of ice recrystallization in low-sugar ice cream formulations [11]. Ultrasonication, a non-thermal processing technique, induces conformational unfolding of proteins, exposing their hydrophobic cores and creating favorable conditions for the formation of protein fibrils [12]. Zhang et al. [13] suggested that ultrasonication treatment facilitated the assembly of soy protein fibrils more effectively than cold plasma or NaCl treatment.

Despite recent advancements, research on protein fibrils within ice cream remains limited, particularly with respect to oleogel-based ice cream systems. The present study aimed to investigate the physical property and adsorption behavior of WPIF subjected to varying ultrasonication durations (0–12 min). These modified protein fibrils were subsequently incorporated into oleogel-based ice cream formulations. The effects of WPIF on the characteristics of ice cream emulsions were evaluated by analyzing fat crystallization, ice crystallization, fat destabilization, microrheological properties, oral tribology, melting behavior, and so forth. This research highlights the potential to enhance the quality attributes of oleogel-based ice cream through the precise design of its emulsion structure.

## 2 Materials and methods

### 2.1 Materials

WPI was supplied by Fonterra Cooperative Group Ltd. (Palmerston North, New Zealand). Beeswax (melting point 45–65 °C) was provided by Likang Technology Co., Ltd. (Beijing, China). Hydrogenated palm kernel oil (HPKO) was purchased from Yihai Kerry Co., Ltd. (Shanghai, China). Soybean oil was purchased from Yongsheng Industry and Trade Co., Ltd. (Guangzhou, China). Sucrose stearate (S370, HLB = 3) was obtained from Mitsubishi Chemical Co., Ltd. (Tokyo, Japan). Guar gum and sucrose were procured from a local supermarket. Nile red and fluorescein isothiocyanate (FITC) isomers (≥90.0% purity) were purchased from Sigma-Aldrich Chemical Co., Ltd. (St. Louis, MO, USA). Hydrochloric acid (HCl) was supplied by Tianjin Bohai Chemical Co., Ltd. (Tianjin, China). All emulsions were prepared using Milli-Q® water (Millipore Co., Ltd., Darmstadt, Germany).

### 2.2 Preparation of WPIF

WPI powder was dissolved in water and stirred at room temperature for 2 h. Subsequently, the solution was incubated at 4 ℃ overnight to ensure complete hydration. Thereafter, the pH was adjusted to 2.0 with HCl. The solution was heated at 90 ℃ for 5 h, followed by rapid cooling to 25 ℃ using an ice bath to prepare WPIF [14]. The prepared WPIF was subjected to ultrasonic treatment for predetermined intervals of 0, 4, 8, and 12 min at a power level of 500 W and a frequency of 20 kHz, employing an Ultrasonic Cell Disruptor SCIENTZ-ⅡD (Ningbo Scientz Biotechnology Co., Ltd., China).

### 2.3 Protein properties

#### 2.3.1 Transmission electron microscopy (TEM)

The diluted protein solution was dripped onto a special copper mesh and allowed to dry naturally. Afterwards, a droplet of 2% phosphotungstic acid was placed onto the copper mesh and incubated for negative staining. The morphology of the sample was subsequently analyzed utilizing TEM (Tecnai G2 F20, FEI, USA) at 200 kV.

#### 2.3.2 Zeta-potential

The zeta-potential of the protein solution was determined utilizing a Zetasizer Nano ZS (Malvern Instruments, UK). All measurements were performed in triplicate under a controlled temperature of 25 ℃.

#### 2.3.3 Surface hydrophobicity (H_0_)

The surface hydrophobicity of protein was determined utilizing a Varioskan Flash multimode plate reader (Thermo Fisher Scientific, USA). Samples were prepared by diluting the protein to concentrations ranging from 0.01% to 0.5% (w/v) using 0.01 M phosphate buffers. Subsequently, 50 µL of 1-anilinonaphthalene-8-sulfonic acid (ANS) (8.0 mM) was introduced into 4 mL of each sample, and the mixtures were incubated in the dark for 3 min. Fluorescence intensity measurements were conducted using an excitation wavelength of 390 nm and an emission wavelength of 470 nm [15]. The initial slope derived from the plots of fluorescence intensity versus protein concentration (mg/mL) was employed as an indicator of H_0_.

#### 2.3.4 Intrinsic fluorescence

The emission spectra of the protein samples within the wavelength range of 300 to 450 nm were measured using an FS5 Fluorescence Spectrometer (Edinburgh Instruments Ltd., UK) at ambient temperature. Measurements were performed with a 1 cm path length cell, an excitation wavelength of 280 nm, and slit widths of 5 nm for both excitation and emission [16].

#### 2.3.5 Interfacial tension and dilatational modulus

The adsorption characteristics of WPI and WPIFs, which displayed distinct morphologies at the oil–water interface, were examined using an interfacial rheometry (DSA30S, Krüss GmbH, Hamburg, Germany). Temporal changes in interfacial tension were assessed via the pendant drop technique. Specifically, a droplet of medium-chain triglyceride (MCT) oil, with a volume of approximately 30 μL, was generated at the tip of a U-shaped needle (inner diameter 0.998 mm) immersed in the aqueous phase within a quartz cuvette. Equilibrium interfacial tension values were obtained by fitting the droplet profile to the Young-Laplace equation. Additionally, the dilatational rheological properties of the interface were assessed using the oscillating drop technique, which involves analyzing the profile of a droplet formed at the interface subjected to periodic deformation. The dilatational modulus was evaluated through the application of sinusoidal oscillations with strain amplitudes varying between 0% and 40%, and frequencies ranging from 0.01 Hz to 0.2 Hz.

#### 2.3.6 Quartz crystal microbalance with dissipation monitoring (QCM-D)

Prior to conducting measurements, the gold sensors were cleaned with a solution consisting of ultrapure water, 25% ammonia, and 30% hydrogen peroxide mixed in a volumetric ratio of 5:1:1, maintained at 75 ℃ for 10 min. The sensors were thoroughly rinsed with ultrapure water and subsequently dried. QCM-D experiments were carried out utilizing a Q-sense E4 instrument (Biolin Scientific, Sweden). The experiment was started by ultrapure water into the QCM-D chamber until a stable baseline was obtained for the frequency and dissipation. The flow rate was maintained at a constant 0.1 mL/min throughout the experiments, which were performed at a controlled temperature of 25 ℃. To investigate protein adsorption and the interactions between protein and emulsifier, a 0.1% protein solution followed by a 0.1% sucrose stearate solution were sequentially introduced to the sensor, with rinsing steps implemented between each introduction. Finally, the ultrapure water was used to rinse the surface again to rinse away unbound or weakly bound emulsifiers. Data analysis was performed using the QSense Dfind software [17].

### 2.4 Preparation of oleogel and ice cream

A mixture comprising 7 wt% beeswax and soybean oil was heated to 65 ℃ and stirred until the beeswax was fully dissolved. The resulting molten mixture was then solidified at 4 ℃ for 24 h to produce an oleogel [18]. Ice cream emulsions were prepared with fixed ingredient ratios comprising 14 wt% sucrose, 10 wt% fat (either HPKO or OG), 4 wt% protein, 0.3 wt% sucrose stearate, and 0.25 wt% guar gum. The raw materials were mixed using an RW20 digital stirrer (IKA Co., Staufen, Germany) at 700 rpm for 30 min at 60 ℃. The resulting coarse emulsions were then subjected to two-stage high-pressure homogenization at 200 bar and 100 bar, respectively, employing an AH-2010 homogenizer (ATS Engineering Inc., Ontario, Canada). The freshly prepared ice cream emulsions were rapidly cooled and subsequently aged overnight at 4 ℃. The emulsions were processed in a scraped-surface batch ice cream freezer (IC6308C, Petrus Electric Co., Ltd., Foshan, China) for freezing and whipping. Finally, the ice cream was hardened at −18 ℃ for 24 h prior to further analysis.

### 2.5 Emulsion properties

#### 2.5.1 Fat crystal and ice crystal

Fat crystals within the coarse emulsion were examined utilizing polarized light microscopy (LV100-PO; Nikon Corporation, Tokyo, Japan). For sample preparation, the emulsion was stirred at 8000 r/min for 1 min, serving as an alternative to high-pressure homogenization. Subsequently, a 15 μL aliquot of the emulsion was evenly applied onto a glass slide, and the sample was stored at 4 ℃ for 24 h prior to microstructural analysis. To characterize the distribution of ice crystals within the emulsion, the growth morphology of ice crystals was observed utilizing a Linkam LTS120 thermoelectrically controlled cooling apparatus (Linkam, Surrey, UK). This system comprises a temperature controller enabling precise regulation, a cooling chamber, a water pump for heat dissipation, and a microscope equipped with a camera for observation. The experimental procedure employed a Peltier cooling stage, which facilitates temperature modulation within a range of 120 ℃ to −40 ℃ with an accuracy of ± 0.01 ℃ [19].

#### 2.5.2 Droplet size and fat destabilization

The particle size distribution and the average particle diameter (D_4,3_) of the emulsions were measured by laser light scattering utilizing a Mastersizer 2000 (Malvern Instruments Ltd., Worcestershire, UK). Prior to analysis, all emulsions were diluted at a 1:100 (v/v) ratio with either deionized water or a 2% SDS solution to facilitate differentiation between the flocculation degree (FD) and partial coalescence degree (PCD) of the emulsions. The FD and PCD values were calculated using the following equations:

(1) $$FD\;\left(\%\right)=\frac{D_{4,3(water)}-D_{4,3(SDS)}}{D_{4,3(SDS)}}\times 100$$

(2) $$PCD\;\left(\%\right)=\frac{D_{4,3(SDS)WE}-D_{4,3(SDS)E}}{D_{4,3(SDS)E}}\times 100$$

where D_4,3(water)_ and D_4,3(SDS)_ are the volume-weighted diameters measured in aqueous and SDS solutions, respectively. D_4,3(SDS)E_ and D_4,3(SDS)WE_ correspond to the volume-weighted diameters of aged emulsions and ice cream emulsions, respectively, as evaluated in the presence of the SDS solution.

#### 2.5.3 Microrheological test

The microrheological properties of the emulsions were measured utilizing diffusing wave spectroscopy (DWS) performed with a microrheolaser instrument (Rheolaser Master, Formulaction, Toulouse, France). The emulsions were subjected to uniform agitation and subsequently placed into the chamber of the instrument. Data acquisition and subsequent analysis were conducted utilizing the Rheosoft Master 1.4 software, which facilitated the determination of key parameters including the mean square displacement (MSD), macroscopic viscosity index (MVI), and elasticity index (EI).

### 2.6 Ice cream characterization

#### 2.6.1 Overrun

The overrun was quantified following the method described by Liu et al. [20]. An aluminum container of identical volume was employed to measure the weights of both the ice cream mix and the finished ice cream. Subsequently, the overrun was calculated using the following formula:

(3) $$Overrun\;\left(\%\right)=\frac{Weight\;of\;mix-weight\;of\;ice\;cream}{Weight\;of\;ice\;cream}\times 100$$

#### 2.6.2 Hardness

The hardness of the prepared ice cream was evaluated using the TA-XT2i Texture Analyzer (Stable Microsystems, Surrey, UK) through texture profile analysis (TPA). The analysis was conducted under the following conditions: a probe diameter of 36 mm, both the initial and test speeds set at 2 mm/s, a penetration depth of 20 mm, and a trigger force of 1 N [21]. The test was performed at ambient temperature and humidity (20–25 °C and 50%–65%, respectively), and the whole process was finished within 20 s.

#### 2.6.3 Oral tribology

The tribological characteristics of ice creams were evaluated utilizing a stress-controlled HAAKE Mars rheometer (Thermo Electron GmbH, Karlsruhe, Germany). To simulate the oral processing of emulsions, the contact interface comprised a three-ball-on-plate arrangement, constructed from commercial polydimethylsiloxane balls and plates (Sylgard 184, Dow Chemical, Midland, MI, USA), housed within a cone-shaped measurement cell. Prior to lowering the probe to the measurement position, 0.6 mL of the sample was introduced into the cell. A constant normal force of 1 N was applied at 25 °C, and the friction coefficient was recorded across sliding speeds ranging from 0.1 to 300 mm/s at logarithmic intervals [22].

#### 2.6.4 Melting behavior

The melting behavior was investigated following the method described by Alvi et al. [23] with some modifications. Equal volumes of ice cream samples were melted at ambient temperature and subsequently placed on a 2.5 mm stainless steel wire mesh filter, with pore dimensions of 10 × 10 mm. Throughout the melting assessment, the ability of the samples to preserve their initial form was methodically assessed through periodic visual observations. Photographic documentation of the samples was conducted at 30 and 60 min to record morphological changes over time.

#### 2.6.5 Bubble distribution

Micrographs of the ice cream emulsions were acquired using a confocal laser scanning microscope (CLSM; LSM 800, Carl Zeiss Inc., Oberkochen, Germany). Sample weighing 800 g was treated with 1 mL of FITC solution (0.1 wt%) and 2 mL of Nile red solution (0.1 wt%). Imaging of the stained ice cream samples was performed subsequent to the whipping and freezing processes. Observations were performed using a 63 × oil immersion objective lens. FITC and Nile red were excited by Argon and Helium-Neon lasers at excitation wavelengths of 488 nm and 633 nm, respectively, with emission detection ranges set at 500–588 nm for FITC and 640–755 nm for Nile red.

### 2.7 Statistical analysis

All experiments were conducted in triplicate, and the data are presented as the mean ± standard deviation. Statistical analyses were performed using GraphPad Prism version 9.0. For comparisons between two groups, a two-tailed Student’s *t*-test was employed. Statistical significance was defined as *p* < 0.05.

## 3 Results and discussion

### 3.1 Protein properties

#### 3.1.1 TEM images and zeta-potential

The impact of different ultrasonication durations on the morphological and physical properties of WPIF was examined. As illustrated in Fig. 1A, significant alterations in WPIF length were observed. Under conditions of low pH and elevated temperature, proteins undergo hydrolysis into peptides, which subsequently self-assemble into slender and flexible fibrils (1014.89 ± 69.91 nm). Following ultrasonic treatment of WPIF, the fibrils were observed to fragment. Specifically, as the ultrasonication duration increased from 0 to 8 min, the fibrils gradually shortened. An increase in ultrasonication duration corresponded with a decrease in fibril length. After a treatment duration of 4 min, the length of WPIF was 629.71 ± 47.27 nm. An ultrasonication period of 8 min was identified as optimal, promoting the conversion of linear aggregates into shorter linear structures (225.88 ± 22.75 nm). Nevertheless, extending the ultrasonication duration to 12 min resulted in slight aggregation of WPIF (542.39 ± 27.41 nm), which may be attributed to the enhancement of Brownian motion with extended ultrasonication, thereby enabling the reaggregation of fibrils [24]. These larger aggregates impeded their movement to the interface and may introduce steric hindrance, thereby limiting further adsorption processes.

**Fig. 1:**
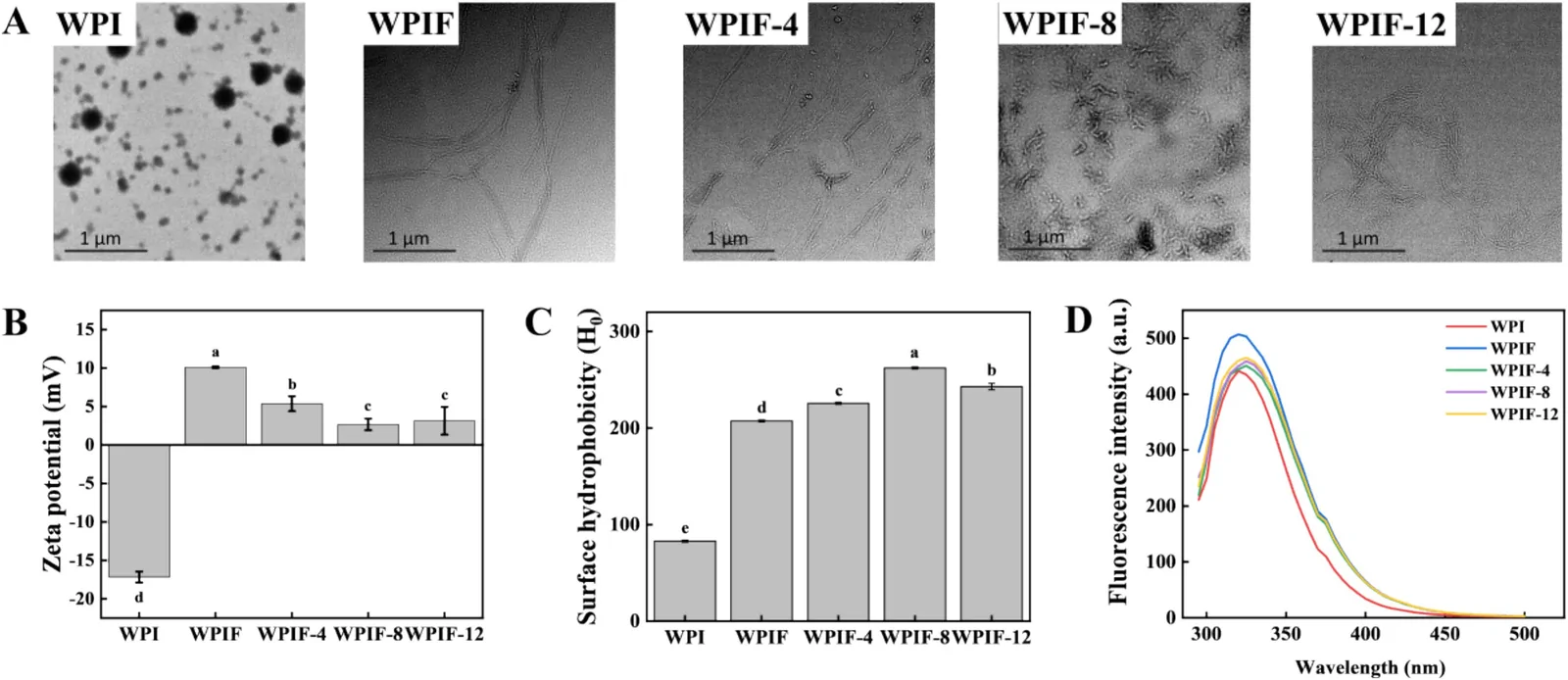
(A) Transmission electron microscopy (TEM) images, (B) zeta-potential values, (C) surface hydrophobicity, and (D) endogenous fluorescence spectra of whey protein isolate (WPI) and WPI fibrils (WPIFs) with different ultrasonication durations. Columns with different letters (a, b, and c) are significantly different (*p* < 0.05).

Fig. 1B illustrates the zeta-potential of the proteins. An elevation in the zeta-potential of WPIF was observed, suggesting the assembly of positively charged building blocks in fibrosis. Furthermore, this increase in zeta-potential was indicative of a stable system and enhanced dispersion of the fibril solution. The observed variations in zeta-potential may be attributed to differences in the “stress response” and the unfolding conformational changes of WPIF following different ultrasonication durations [25].

#### 3.1.2 Surface hydrophobicity

The H_0_ value represents alterations in the hydrophobic groups present on the protein surface, which are critical to various protein functions such as emulsification, foaming, and gelation [16]. As depicted in Fig. 1C, the surface hydrophobicity of WPIF was markedly higher than that of WPI. This increase can be attributed to the unfolding of protein molecules during heat treatment, which exposed additional hydrophobic regions. Perović et al. [26] demonstrated that heating beyond the denaturation temperature induces partial unfolding of the protein structure, thereby revealing hydrophobic groups on the protein surface. Furthermore, the hydrolysis of proteins into low molecular weight peptides may also expose the hydrophobic groups [27].

The assembly of the ordered fibril structure in WPIF is primarily driven by hydrophobic interactions [28]. With increasing ultrasonic treatment time, the H_0_ values of WPIF significantly increased, which can be explained by the unfolding of proteins and the consequent exposure of hydrophobic residues originally sequestered within the protein molecule, facilitated by ultrasonic cavitation and physical shear forces. This structural modification enhanced the protein’s emulsifying capacity. However, when the ultrasonic treatment duration was extended to 12 min, a decrease in H_0_ was observed. This reduction was likely due to the reaggregation of hydrophobic groups through hydrophobic interactions induced by ultrasound, leading to a decrease in overall protein surface hydrophobicity.

#### 3.1.3 Intrinsic fluorescence

The intrinsic fluorescence of proteins serves as a reliable indicator of alterations in the tertiary structure of protein conformation. WPI demonstrated fluorescence emission with a maximum peak at 330 nm (Fig. 1D), consistent with the findings reported by Ban et al. [29]. In the present study, acid-heat treatment resulted in an increase in the intrinsic fluorescence intensity of WPI, accompanied by a slight red shift, suggesting modifications in the microenvironment surrounding the tryptophan residues within the protein. Notably, the WPIF-8 sample exhibited an enhanced fluorescence intensity, which may be attributed to the increased exposure of hydrophobic amino acid residues on the protein surface following ultrasonic treatment.

The maximum emission wavelength (λ_max_) remained constant regardless of the duration of ultrasonic treatment. WPIF-12 demonstrated a reduced fluorescence intensity compared to WPIF-8, which was consistent with the observed trends in H_0_ values. Consistent with these findings, Li et al. [30] reported that extending ultrasonic treatment to 15 min resulted in lower fluorescence intensity relative to a 10-minute treatment. This decrease may be attributed to the formation of protein aggregates upon excessive ultrasonication, which led to the sequestration of hydrophobic residues within the protein’s interior.

#### 3.1.4 Interfacial properties

The surface activity of proteins enables their rapid adsorption at liquid interfaces, leading to a reduction in interfacial tension [31]. As shown in Fig. 2A, WPIF exhibited lower interfacial tension than WPI, and its accelerated reduction was attributed to protein unfolding at the interface. This observation was consistent with the zeta-potential results. Upon adsorption at the oil–water interface, fibrils attained interfacial saturation more rapidly than individual globular proteins via fibril entanglement. The adsorption of these fibrils resulted in a decrease in interfacial tension, which can be ascribed to the deformation of the fluid interface caused by lateral attractive capillary forces acting between the fibrils [32]. With the extension of ultrasonic treatment duration to 4 or 8 min, the WPIF experienced fragmentation into shorter fibrils (as shown in TEM images), which demonstrated enhanced adsorption kinetics at the oil–water interface compared to the original WPIF. In contrast, WPIF-12 showed a moderate increase in interfacial tension, which may be attributed to the development of a thicker adsorption layer formed by the aggregated short fibrils compared to the more uniformly dispersed fibrils. Interfacial tension is expected to be a primary determinant of foamability, with a more rapid decrease indicating an increased rate of protein adsorption and enhanced stabilization of air bubbles against coalescence [33].

**Fig. 2:**
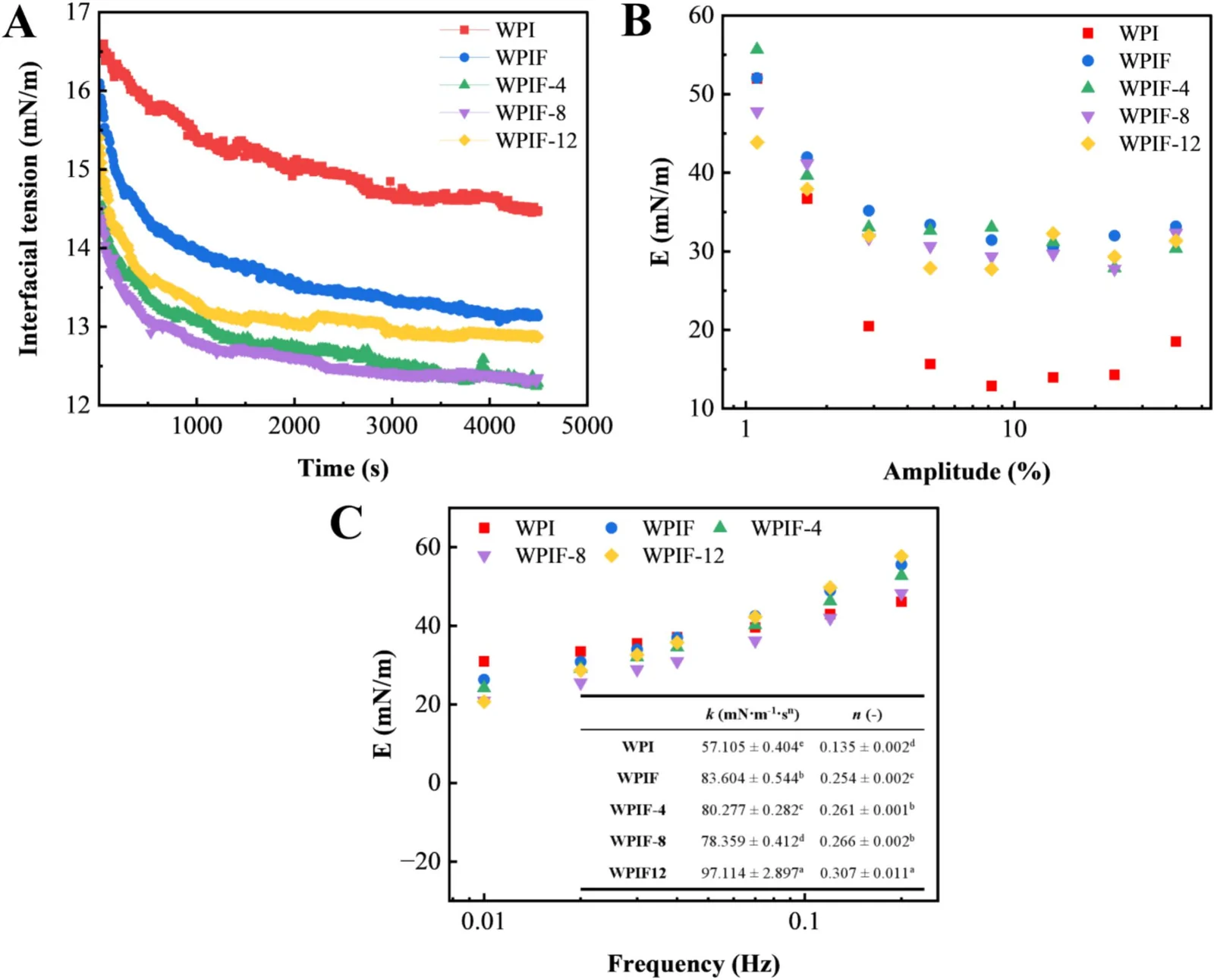
(A) Interfacial pressure of proteins at the oil–water interface and the variations in the complex dilatational modulus with respect to (B) amplitude and (C) frequency. Different superscript letters in the same column of Table indicate are significantly different (*p* < 0.05).

Fig. 2B presents the composite modulus of various samples measured across different amplitudes. The modulus of the modified WPIF at the oil–water interface was significantly higher than that of WPI. Furthermore, the *E* values of the ultrasonically disrupted WPIFs were consistently lower than those of the non-ultrasonicated WPIF, suggesting that fibril length influenced the mechanical strength of the interfacial film. Fig. 2C illustrates the variation of the composite modulus of the protein fibers as a function of frequency, which can be effectively characterized by the power-law model (refer to the data in the table). In comparison to WPI, the ultrasound-treated WPIFs exhibited relatively higher values of *k* and *n*, suggesting an improvement in the interfacial strength of the protein fibrils. The long fibril (WPIF) displayed a lower *n* value than the shorter fibrils (WPIF-4 and WPIF-8) subjected to ultrasound treatment, indicating a greater structuring degree of WPIF at the oil–water interface. Among the samples, WPIF-12 demonstrated the highest *k* and *n* values, and its modulus varied markedly with frequency changes, thereby hindering the development of a stable interfacial film.

#### 3.1.5 Adsorption behavior

QCM-D was employed to investigate the changes in frequency shift (Δ*F*) and dissipation shift (Δ*D*) associated with the adsorption of proteins in the presence of sucrose stearate (S370), as illustrated in Fig. 3. The introduction of proteins induced an initial rapid decline in Δ*F*, which subsequently stabilized at a relatively constant level. This behavior is attributed to the successful adsorption of protein molecules onto the sensor surface. Upon saturation of the available adsorption sites, the interface became fully occupied by protein, resulting in minimal further changes in Δ*F*. Notably, the WPIF sample exhibited a greater adsorption capacity (Δ*F* = −29.9 Hz) compared to WPI. This observation suggests that emulsions stabilized by WPIF are likely to demonstrate enhanced stability.

**Fig. 3:**
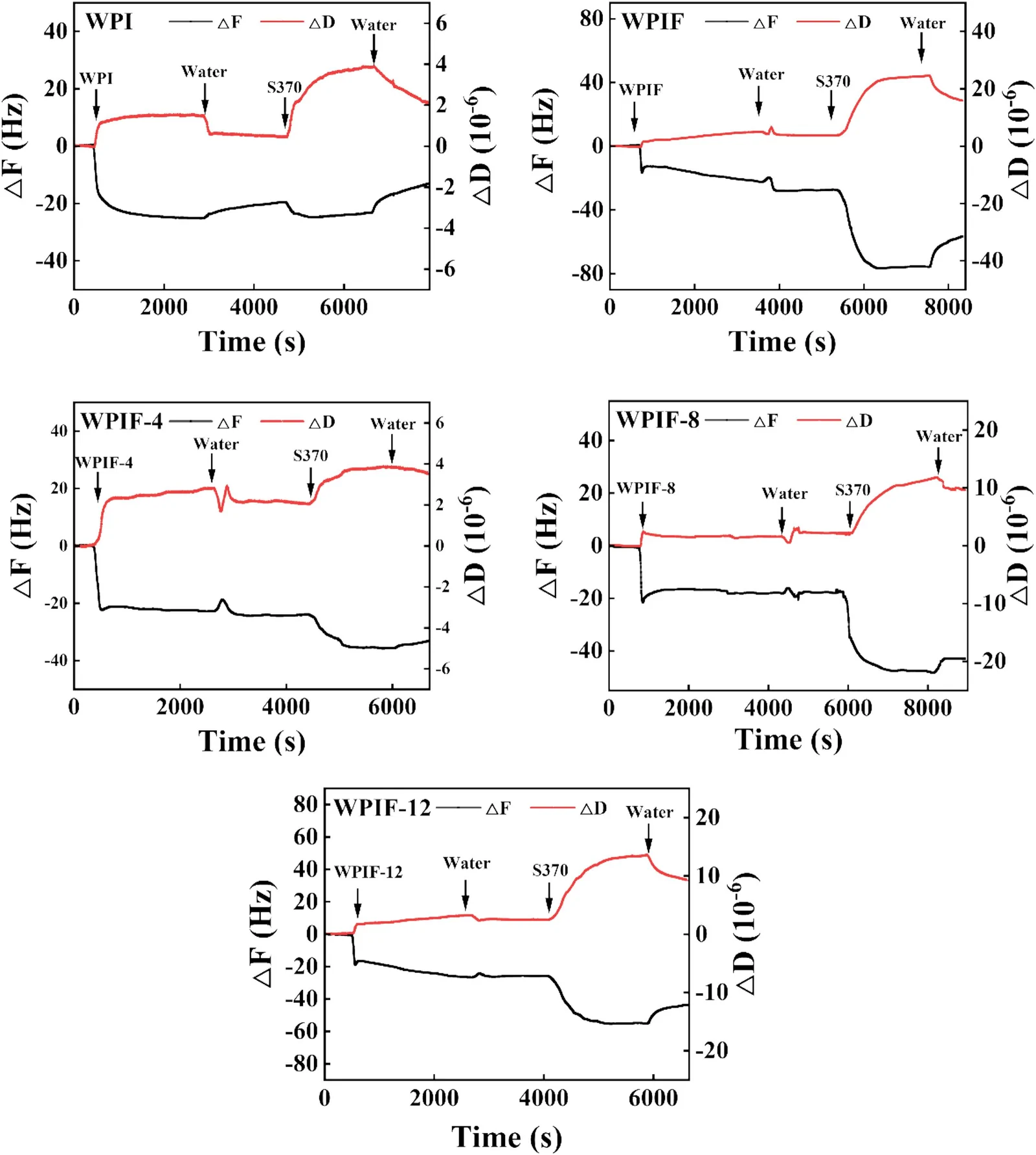
The changes in frequency shift (Δ*F*) and dissipation shift (Δ*D*) of the protein and S370 during the adsorption process at the oil–water interface.

At the beginning of the rinsing phase, the observed increase in Δ*F* was attributed to the rapid desorption of loosely bound or non-adsorbed protein molecules [8]. This increase was notably more pronounced for WPI than for WPIF, suggesting that WPI exhibited weaker attachment to the interface. Subsequently, the introduction of S370 into each system led to a rapid and significant reduction in Δ*F* values across all systems, followed by a gradual stabilization. This behavior indicated the adsorption of S370 onto the protein-coated chip surface. Moreover, S370 demonstrated the capacity to interact with all protein-coated surfaces and could not be entirely removed from the sensor by washing. Notably, the system containing WPIF exhibited the lowest Δ*F* value, implying the formation of the thickest interface layer of WPIF and emulsifier S370 [34].

The temporal trend of Δ*D* mirrored that of Δ*F* but in the opposite direction. The increased energy dissipation measured on the quartz crystal surface is attributable to the formation of a soft, hydrated, and viscoelastic emulsifier layer. Layers exhibiting Δ*D* values exceeding 1 × 10^-6^ are classified as viscoelastic [17]. Among the samples, WPIF demonstrated the greatest adsorption thickness following adsorption and desorption procedures. However, ultrasonic treatment led to a reduction in the quality of the adsorption layer formed by WPIF and S370, indicating a weakening of their interaction. Generally, a thicker and more rigid adsorption layer surrounding oil droplets within an emulsion enhances spatial repulsive forces while diminishing van der Waals attractions, thereby improving resistance to aggregation [35].

### 3.2 Emulsion properties

#### 3.2.1 Fat crystal and ice crystal

Fig. 4 illustrates the presence of fat and ice crystals in emulsions containing different fat phases. In comparison to the WPI-HPKO emulsion, other OG-based samples demonstrated more pronounced birefringent crystals. The alkyl chains of beeswax within the fat globules interact with triglyceride molecules, promoting the formation of N-type and hard crystals in OG-based emulsions [36]. Achieving a smooth ice cream texture necessitates that most ice crystals remain below approximately 50 μm in size; notably, smaller ice crystals ranging from 10 to 20 μm contribute to a smooth and creamy final product [37]. Upon substituting HPKO with OG, fat crystals exhibited needle-like or plate-like morphologies, while ice crystals became irregular and larger. Among all samples, the WPI-OG emulsion presented the largest fat and ice crystal sizes, accompanied by poor uniformity and low distribution density within the emulsion.

**Fig. 4:**
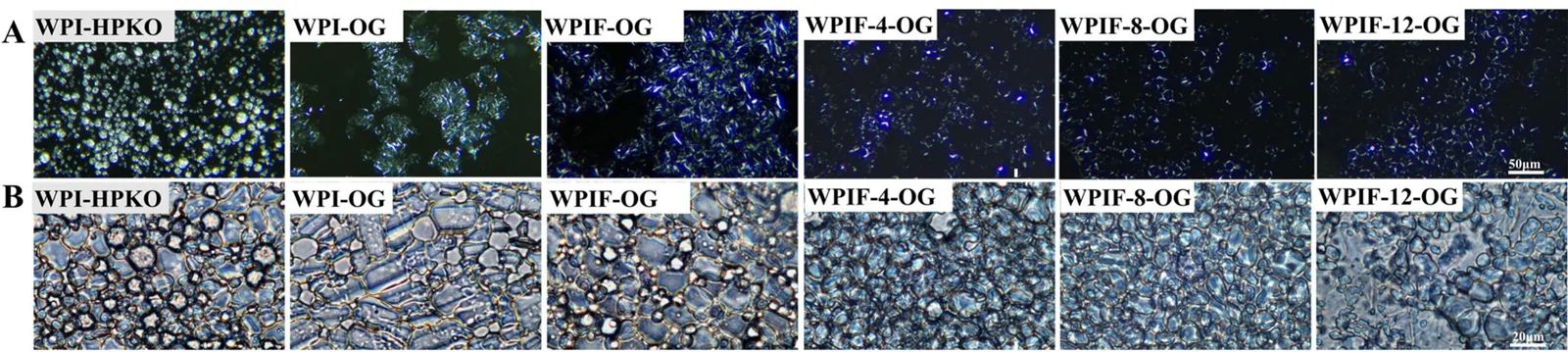
(A) Fat crystal and (B) ice crystal of emulsions.

Water-holding capacity constitutes a key parameter for assessing proteins utilized in ice cream formulations, primarily due to its critical function in regulating ice crystal formation within the final product. The development of small and uniform ice crystals at low temperature is essential for preserving the structural stability of the ice cream and for modulating its texture. In the present study, ice creams formulated with WIPFs exhibited comparatively smaller fat and ice crystal sizes, which can be attributed to the enhanced water-holding capacity of the protein, thereby reducing crystal dimensions. Gao et al. [11] reported that WPIFs serve as ideal amphiphilic agents capable of stabilizing oil–water and air–water interfaces, effectively inhibiting ice recrystallization. The WPIF-8-OG sample demonstrated the smallest fat and ice crystals with the most uniform distribution. In contrast, the WPIF-12-OG sample displayed noticeable aggregation of fat globules and larger ice crystals. These results indicated that suitable ultrasonic treatment may improve the water-holding capacity of the protein, thereby facilitating the formation of a higher quantity of smaller ice crystals. This observation is consistent with findings reported by Yang et al. [38].

#### 3.2.2 Microrheology

Microrheological techniques can be employed to assess the Brownian motion of emulsion droplets and to investigate the interactions between particles. As illustrated in Fig. 5A, the mean squared displacement (MSD) curves of the emulsions exhibited nonlinear behavior, suggesting that these systems possess viscoelastic properties and that the droplet mobility is constrained by interactions within the droplet network [35]. The relaxation time observed in the plateau region for the WPIF-OG samples was longer compared to those of the WPI-HPKO and WPI-OG samples, which may be attributed to increased emulsion viscosity and the reinforcement of droplet dynamics through fat network binding [4]. These findings implied that ultrasonic treatment applied for 4 or 8 min potentially facilitated intermolecular entanglement, thereby promoting the formation of an enhanced viscoelastic network with an improved response to oscillatory deformation [39].

**Fig. 5:**
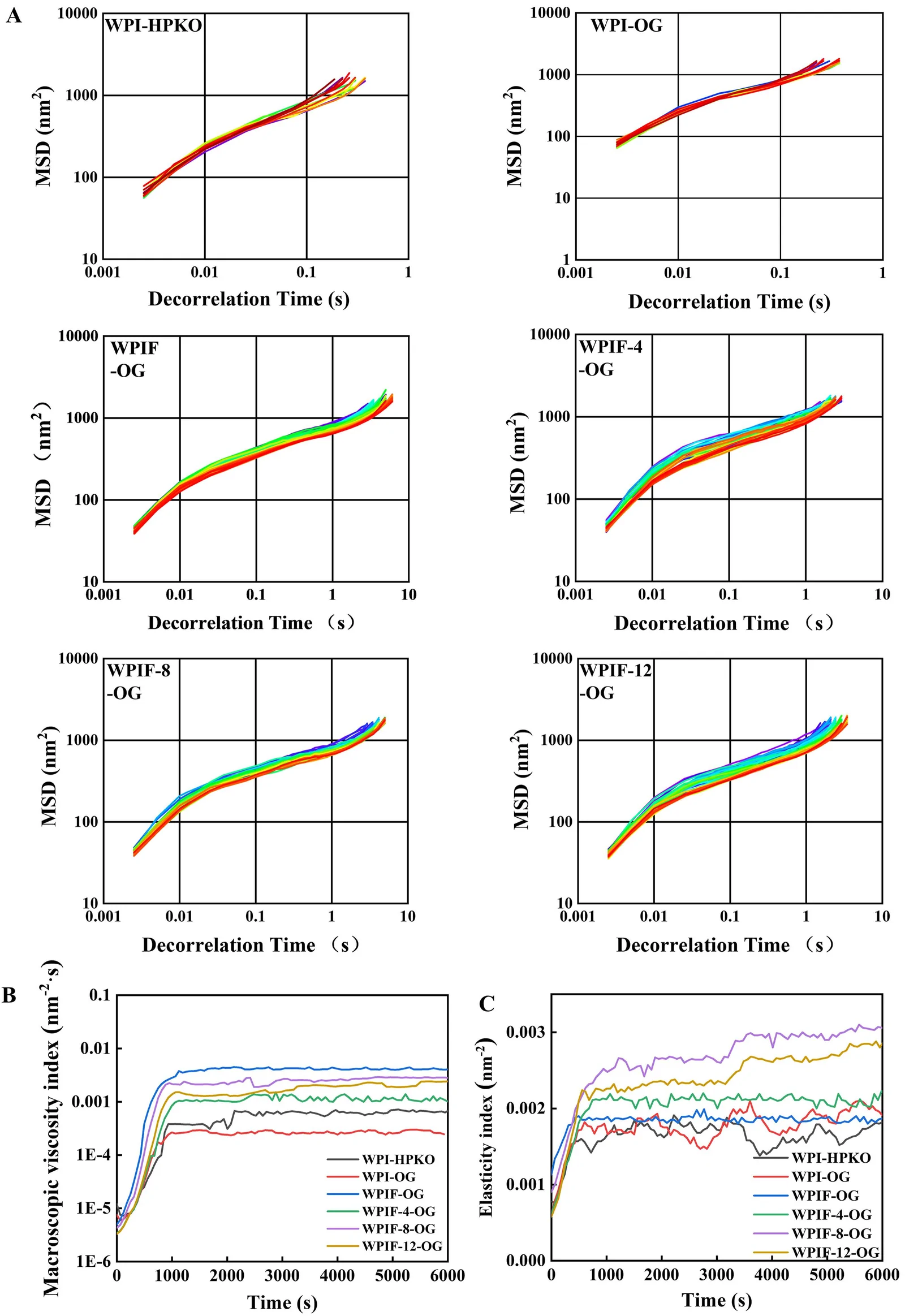
(A) Mean squared displacement (MSD) curves, (B) macroscopic viscosity index (MVI), and (C) elasticity index (EI) of emulsions.

The macroscopic viscosity index (MVI) and elasticity index (EI), derived from MSD curves, serve as quantitative descriptors of the viscosity and elasticity characteristics of emulsion samples. The MVI correlates with the rheological behavior of samples under zero shear conditions and is inversely proportional to the velocity of colloidal particles. The EI corresponds to the reciprocal of the displacement distance that particles traverse prior to interacting with a particle network. Typically, increases in MVI and EI are attributed to heightened interdroplet resistance [40]. As illustrated in Fig. 5B, the WPI-HPKO emulsion exhibited a higher MVI value compared to WPI-OG, potentially due to the elevated saturated fatty acid content in HPKO, which influenced the molecular arrangement during triglyceride crystallization and promoted the formation of a more robust network structure. Additionally, the WPIF-OG sample, characterized by strong water-holding capacity, may impede water flow, thereby increasing the viscosity of the emulsions. The elevated viscosity and the internal droplet network reduce the dynamic activity of the emulsion, which likely contributes to enhanced emulsion stability. Specifically, WPIFs of ultrasonic treatment resulted in reduction in the overall viscosity of the emulsions.

Moreover, the EI demonstrated a significant increase over time, eventually attaining a state of relative equilibrium (Fig. 5C). This observation suggested the formation and stabilization of a three-dimensional network among emulsion droplets [41]. An elevated EI value is indicative of enhanced network strength. In comparison to the WPI-HPKO emulsion, the OG samples exhibited higher EI values, implying that the incorporation of oleogels contributed to the formation of more compact gel networks. The elastic modulus is associated with the microstructure as well as the crystalline characteristics of the system. Ultrasound treatment led to an increase in EI, indicating the development of an interfacial film at the oil–water interface with greater elasticity, which in turn enhanced resistance to droplet movement and delayed flocculation or aggregation [41]. Among the samples, WPIF-8-OG displayed the highest EI value, corresponding to the densest network, whereas WPIF-12-OG showed a reduction in EI. This decrease may be attributed to the reduced ability of aggregated protein fibrils to establish a stable interfacial protein layer, potentially resulting in phase separation and droplet aggregation.

#### 3.2.3 Particle size distribution and fat destabilization

A certain degree of instability in fat globules has been found to facilitate the development of ice cream texture during the whipping and freezing processes [42]. To assess the stability of emulsions, measurements of particle size distribution and fat destabilization indices (FD and PCD) were conducted. As illustrated in Fig. 6A, each emulsion exhibited two or three distinct distribution peaks. The initial peak corresponded to individual fat globules with sizes ranging from 0.1 to 10 μm, while the subsequent peaks at larger diameters indicated the presence of fat aggregates formed through partial coalescence [43]. Following the addition of SDS, a slight reduction in particle size was observed, attributed to the dissociation of fat flocculates induced by the SDS solution. Emulsions composed of WPI-OG/WPIF-OG demonstrated larger particle sizes compared to WPI-HPKO emulsions, which was likely due to interactions between the alkyl chains of beeswax and triglyceride molecules, contributing to the formation of larger and more rigid crystals [36]. Furthermore, an increase in particle size was detected in aged emulsions, resulting from fat crystallization at 4 °C that led to the aggregation of larger droplets [4]. Ice cream emulsions exhibited a reduction in particle size, as the whipping and aeration processes generated smaller droplets.

**Fig. 6:**
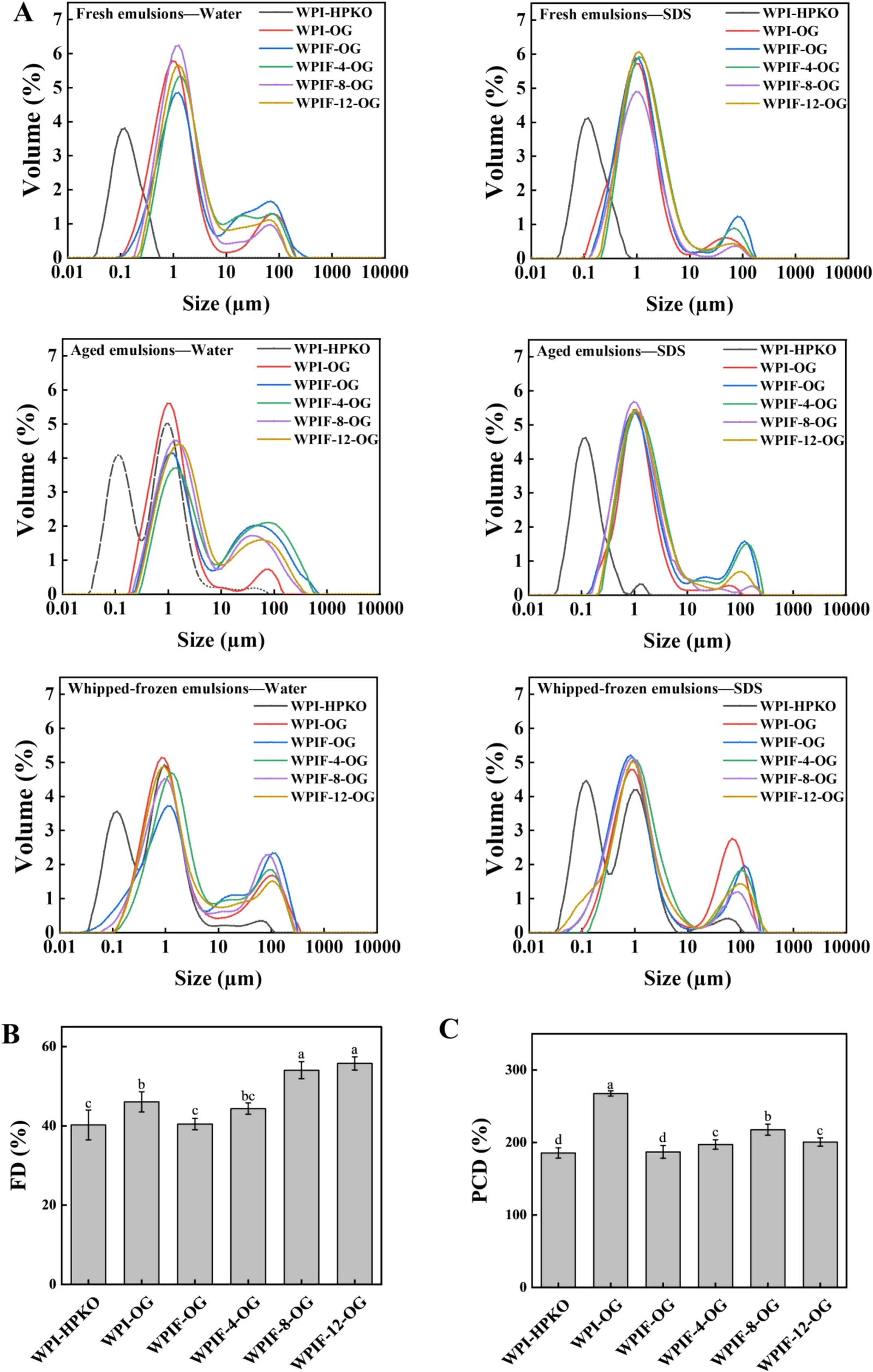
(A) Particle size distributions, (B) flocculation degree (FD), and (C) partial coalescence degree (PCD) of emulsions. Columns with different letters (a, b, and c) are significantly different (*p* < 0.05).

Due to the thermodynamic instability of emulsions, droplet interactions are prone to occur during storage, which are mainly bridge/depletion flocculation and coalescence. The FD value observed in the WPI-OG sample was greater than that measured in the WPI-HPKO emulsion, as illustrated in Fig. 6B. This destabilization was primarily attributed to the formation of larger fat crystal sizes within OG-based emulsions, which consequently increased the protrusion length at the surface of fat globules [44]. Furthermore, the elevated FD values observed in the WPIF emulsions may result from an increase in protein-binding clusters formed during the whipping and freezing processes [45]. Acid-thermal treatment has been shown to modify the hydrophobicity and flexibility of proteins, thereby influencing their aggregation behavior and interfacial properties at the oil–water interface.

During the ice cream freezing process, the fat present in the mixture undergoes partial coalescence or destabilization as a result of emulsifier activity, air incorporation, ice crystallization, and intense shearing forces within the freezer. This partial coalescence of fat is essential for developing the structure and texture of ice cream, which closely resembles that of whipped cream [46]. As shown in Fig. 6C, emulsions containing OG demonstrated higher PCD values than that with HPKO, which was attributed to the presence of liquid oil in the dispersed phase that wet the connected fat globules, thereby strengthening their interactions [47]. Increased fat destabilization to some extent via partial coalescence positively influenced the elasticity and solidity of ice cream. The rate of coalescence is affected by both the solid fat content (SFC) and the crystallization characteristics of fat. It has been proposed that pH-shifting and ultrasound treatments enhance the functionality of protein fibrils, thereby decreasing the size of emulsion droplets and improving the crystallization behavior [38]. Keerati-u-rai et al. [48] reported the aggregation of oil droplets in emulsions stabilized by soy protein isolate following heat treatment. WPIF-OG emulsions exhibited enhanced resistance to partial coalescence compared to WPI-OG emulsions, which can be attributed to the formation of an interfacial film at the oil–water interface exhibiting higher elasticity in the WPIF-OG samples. It has been demonstrated that fat globules experience partial coalescence, a process affected by the protein composition and the fat crystallization behavior [47]. The excellent coalescence stability of an emulsion can likely be ascribed to the high interfacial adsorption and the formation of a stable viscoelastic interfacial layer [49].

### 3.3 Ice cream characterization

#### 3.3.1 Overrun and hardness

Overrun is influenced by the properties of the whipped emulsion, particularly the degree of partial fat coalescence and the viscosity of the emulsion. Fig. 7A presents the overrun of emulsions containing different fat phases. The formation of a fat crystal separation layer between emulsion droplets lead to the aggregation of fat crystals around air bubbles, serving as a critical factor in bubble stabilization [5]. Among the samples, the WPI-HPKO emulsion demonstrated the highest overrun value (70.54 ± 1.72%). The crystalline nature of beeswax significantly contributed to the oleogel structure within the ice cream, enabling it to mimic the structure-forming properties of solid crystalline fat. The WPI-OG emulsion exhibited a lower overrun of 48.32 ± 0.58% compared to the WPI-HPKO sample. Consistent with these findings, Zulim-Botega et al. (2013) reported that although rice bran wax oleogel droplets displayed certain fat-structuring characteristics, the three-dimensional fat network they formed was comparatively weaker than that of milk fat. Furthermore, the elasticity of interfacial film in WPI-OG sample was not great as that of WPIF-OG, resulting in the bubble expulsion and increased the rate of partial coalescence in the WPI-OG ice cream, thereby compromising bubble stabilization. Collectively, these factors promoted the formation of large, unevenly distributed air bubbles, ultimately yielding ice cream with a structurally weak matrix [8].

**Fig. 7:**
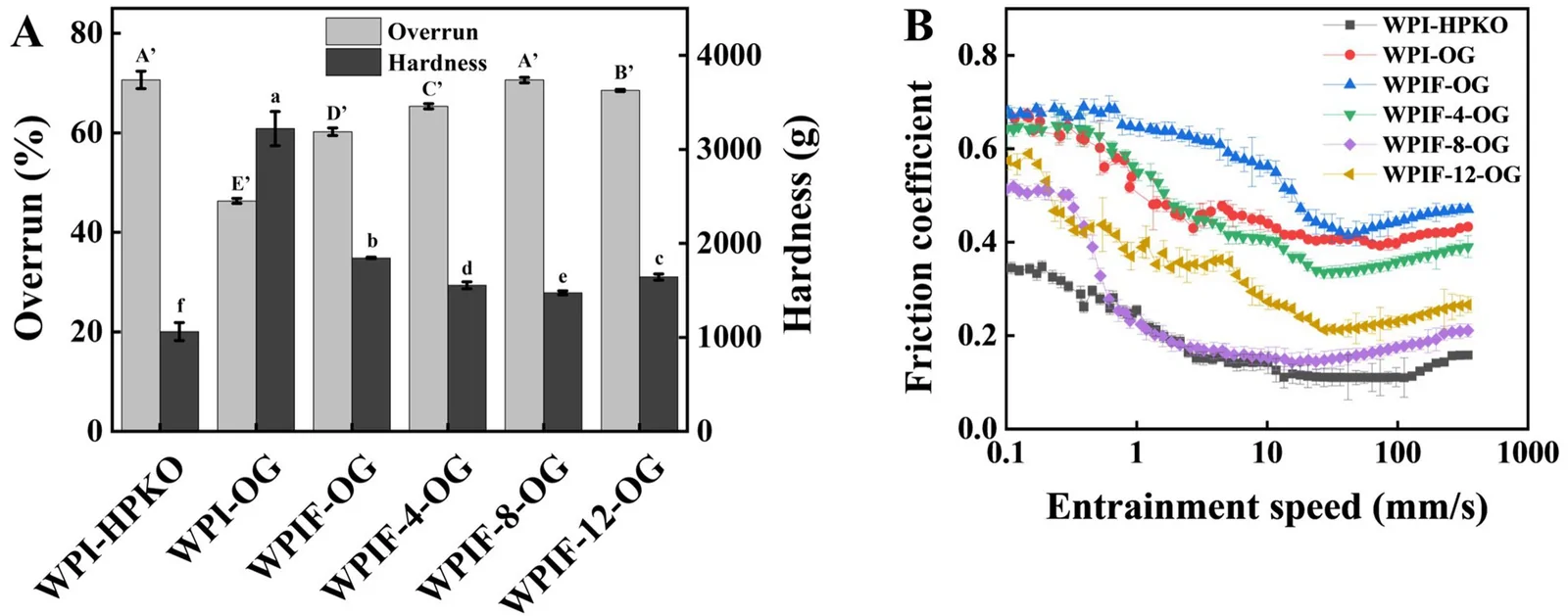
(A) Overrun and hardness, and (B) tribological properties of ice creams. Columns with different letters (a, b, and c) are significantly different (*p* < 0.05).

Significantly, ice cream emulsions stabilized by WPIF exhibited elevated overrun values, which may be attributed to the protein’s influence on the crystallization behavior of the aqueous phase, thereby altering its viscosity. The rapid adsorption of WPIF at the oil–water interface inhibited the aggregation and merging of air bubbles. Furthermore, the modified protein was capable of forming an appropriate network structure between bubbles, facilitating the stabilization of bubbles by fat globules [46]. Increased emulsion viscosity also enhanced the flow resistance of the liquid phase, consequently decelerating foam drainage. These factors collectively contributed to the formation of smaller and more stable air bubbles. Additionally, emulsions stabilized with ultrasonically treated WPIF demonstrated higher overrun values, suggesting that suitable ultrasonic processing of protein fibrils promoted air retention in ice cream stabilized by these fibrils. Hei et al. [8] also stated that protein particles with distinct properties generated different interfacial films, which in turn influenced the microstructure and texture of ice cream.

Hardness serves as a critical parameter for assessing the texture and sensory qualities of ice cream. Factors such as the degree of fat aggregation, ice crystal size, and the proportion of the ice phase collectively influence the hardness of the product [50]. As shown in Fig. 7A, notable differences in hardness were detected across the various ice cream formulations, with the samples containing WPIF-OG demonstrating a more desirable hardness characteristic. Previous research has indicated that the presence of small, uniformly distributed ice crystals combined with a high overrun value enhances the volume of the compressible dispersed phase, thereby reducing resistance to applied force and consequently decreasing the hardness of ice cream emulsions [50]. Ice cream produced with WPI-OG demonstrated increased hardness, attributed to the presence of larger and unevenly distributed air bubbles. This observation is consistent with previous research suggesting that an elevated degree of fat destabilization during freezing promotes the formation of a strong network structure between fat globules and air bubbles, resulting in increased hardness [51]. Notably, the WPIF-8-OG ice cream, which displayed an intermediate hardness (1474.10 ± 21.62 g), contained smaller bubbles with a more uniform distribution, contributing to its desirable texture and overall palatability.

#### 3.3.2 Oral tribology

The texture and mouthfeel of food are essential sensory properties that significantly affect consumer acceptance and preference for various products. By comparing the frictional characteristics of samples through oral tribology, the sensory attributes (such as thickness and smoothness) of fatty products during simulated oral processing can be more effectively described [52]. As illustrated in Fig. 7B, for all samples, an increase in speed resulted in a greater quantity of ice cream being entrained into the gap between the two tribo-pair surfaces. The fat and liquid components of the ice cream began to coat the oral surfaces, leading to partial separation of the interacting surfaces and thereby enhancing lubrication. Consequently, a reduction in the friction coefficient was observed, indicating a transition to the mixed lubrication regime. At even higher speeds, the system entered the hydrodynamic lubrication regime, wherein the liquid components of the food established a continuous lubricating layer.

The WPI-HPKO emulsion exhibited a low friction coefficient, which is consistent with the widely accepted understanding that emulsion foams containing small and uniformly sized bubbles are more effectively entrained, leading to a reduction in friction [53]. In the WPI-OG sample, fat crystallization was found to influence the system, resulting in an enhanced perception of fat and a corresponding increase in the friction coefficient of the ice cream. The WPIF-8-OG sample exhibited exceptional lubricating performance, a phenomenon attributed to the presence of regularly shaped and uniformly dispersed ice crystals within the emulsion. These crystals facilitated the formation of a more effective lubricating layer, thereby decreasing the friction coefficient. This explanation is further corroborated by the result presented in Fig. 4. In contrast, the lubricating properties of lipids in other samples may not be fully manifested. Previous study suggested that emulsion droplets with reduced stability are more prone to destabilization during whipping, which in turn enhances lubrication [54]. Additionally, a finer oral texture, characterized by a creamy mouthfeel, is associated with a higher bubble content within the system. It was observed that excessive ultrasonic treatment of WPIF adversely impacted the smooth and creamy sensation experienced during oral processing. Namely, the WPIF-12-OG sample displayed ice crystals with uneven sizes and distributions, which led to an increased presence of solid particles in the contact area that were either flattened or fractured. This resulted in elevated friction and consequently altered the mouthfeel of the product [52].

#### 3.3.3 Melting behavior and bubble distribution

The viscosity, overrun, and ice crystal size of the ice cream were found to exert a significant impact on its meltdown rate [7]. As depicted in Fig. 8A, the WPI-HPKO sample exhibited a more rapid melting rate compared to the WPI-OG sample. This phenomenon can be attributed to the high SFC of HPKO (> 75%) within the temperature range of 0–10 ℃, followed by a decline between 20–35 ℃, thereby promoting accelerated fat melting. The enhanced melting resistance observed in the WPI-OG sample may be associated with the formation of fat networks, likely resulting from pronounced fat destabilization and the reduced sphericity of air cells [55]. Fat partial coalescence, induced by destabilization, would primarily influence the melting behavior of ice cream by generating a three-dimensional network of fat globules that adhere to and stabilize the surfaces of air bubbles within the ice cream matrix. This network of partially coalesced fat agglomerates not only stabilizes air bubbles but also enhances the flow resistance of the serum phase during melting, thereby reducing the meltdown rate [56]. Compared to the WPI-OG sample, ice creams prepared with WPIF-4 and WPIF-8 demonstrated higher melting rates and more small bubbles (Fig. 8B), which was presumably due to an increased presence of smaller ice and fat crystals within the ice cream microstructure.

**Fig. 8:**
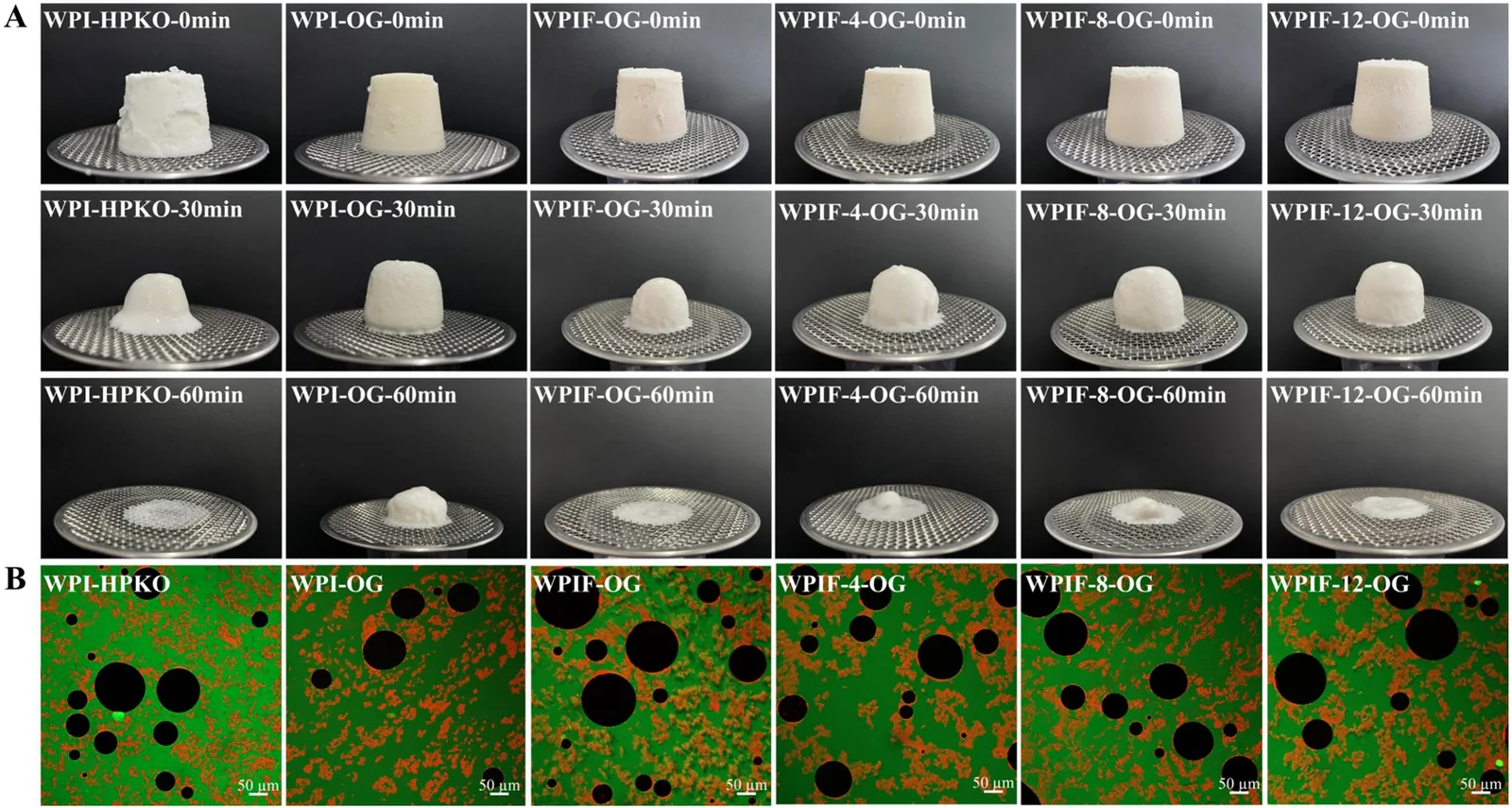
(A) Melting behavior and (B) bubble distribution of ice creams.

## 4 Conclusion

Ultrasonic treatment of WPI induced the formation of fibrillar structures with varying dimensions, which facilitated increased exposure of hydrophobic residues. In oleogel-based emulsion, WPIF subjected to 8 min of ultrasonication resulted in a more uniform and denser distribution of fat and ice crystals, thereby improving emulsion elasticity and promoting fat coalescence. The obtained ice cream demonstrated a high overrun and a low friction coefficient, comparable to those observed in conventional HPKO sample. Additionally, the ultrasonically treated WPIF contributed to favorable melting behavior and bubble distribution in the final product. This study highlights that appropriate ultrasonic modification of WPIF enables precise regulation of oleogel-based ice cream emulsions and substantially improves the structural and quality properties of ice cream. Subsequently, a sensory evaluation of the oleogel-based ice cream will be conducted by trained panelists in conjunction with electroencephalography. The results will be compared with those of commercial products to support the practical application of the ice cream developed in this research. Meanwhile, the expenses associated with ultrasonic processing should also be taken into account.

## CRediT authorship contribution statement

**Ning Liu:** Writing – original draft, Investigation, Formal analysis. **Meng Yan:** Writing – review & editing, Methodology. **Junqi Sha:** Conceptualization. **Kun Zhang:** Writing – review & editing, Conceptualization. **Xiaoting Xue:** Validation, Supervision. **Wenguang Liu:** Software, Methodology. **Xiaomiao Jing:** Validation, Methodology. **Xiaolin Yao:** Funding acquisition, Conceptualization.

## Declaration of competing interest

The authors declare that they have no known competing financial interests or personal relationships that could have appeared to influence the work reported in this paper.
